# Time dependent attachment properties of pollen grains in anemophilous plants tested by the mass centrifugation method

**DOI:** 10.1038/s41598-025-99593-6

**Published:** 2025-04-29

**Authors:** Martin Becker, Stanislav Gorb

**Affiliations:** Department of Functional Morphology and Biomechanics, Zoological Institute, Kiel University, Am Botanischen Garten 1–9, D-24118 Kiel, Germany

**Keywords:** Adhesion, Centrifugation, Pollination, Anemophily, Zoophily, Biophysical methods, Behavioural ecology

## Abstract

The process of pollen release, transfer and capture is the most critical step in reproduction of higher plants and requires several steps of detachment and reattachment of pollen grains to different surfaces. As a response to their specific biotic or abiotic factors, pollen grains have developed a huge variability of size, shape and surface structure, which affects their adhesion properties in a specific manner and contributes to the pollination syndrome of a plant. However, despite decades of research and a great public awareness, these adhesion forces have rarely been measured directly. In the present paper, we used a mass centrifugation setup with glass as a standard substrate, to characterize time dependent adhesion properties of pollen grains from four anemophilous species and compared them to the results of previous studies. Our results show strong differences in adhesion between species studied in fresh and aged state, which can be related to their respective pollination ecology. We discuss the species-specific adhesive properties of pollen grains and highlight methodological aspects, to establish centrifugation method as a standard approach, in order to collect a broad set of quantitative data about adhesion properties of pollen grains and to understand their role in the process of pollination.

**Supplementary Information:**

The online version contains supplementary material available at 10.1038/s41598-025-99593-6.

## Introduction

In order to enable sexual reproduction, plants need to transfer pollen, containing the male gametes, to the female receptive organs of another individual. This transfer process, called pollination, usually requires an additional vector in form of animals or abiotic factors like wind or water^1^. The process of pollination plays an important role in distribution of plants and their adaptation to the environment, as it allows genetic transfer within and also between populations^2^. However, despite several decades of research and a great public awareness, there are still many open questions about the mechanisms underlying successful pollination and how they are affected by surrounding conditions^3^. For example, Zinkl et al. (1999)^4^ found out that despite of the presence of specific recognition molecules on the surface of pollen, which are critical for communication with the female part, the initial contact is rather based on simple physical adhesion and independent of the specific chemical interactions. Therefore, contact mechanics analysis of pollen grains and their physical properties can provide new insights into the adaptations that evolved in a plant species in relation to the specific pollination syndrome.

In the context of a global loss of pollinators, the pollination by animals (zoophily) and especially insects (entomophily) has gained increasing attention during the last years^5^. In general, the great majority of angiosperms depends on animal pollination, while approximately 10% are wind pollinated (anemophilous)^6^. However, among crop plants pollination by animals only reaches about 33% and also many tree species are anemophilous^7^, indicating the importance of wind pollination for agriculture and forestry. From an evolutionary perspective, anemophily is the ancestral pollination syndrome and is in its primary state still present in gymnosperms. In contrast, pollination by animals evolved in angiosperms^3^ and is commonly seen as a major part of their success^8^. However, several species of angiosperms have become secondary anemophilous again, including whole families like grasses^9^, but also minor groups within prominent entomophilous families^10^. In addition, several species are pollinated by both, wind and insects (ambophily), representing a wide spectrum of intermediate stages^11^.

As a consequence of millions of years of evolution, pollen grains of every plant species show specific adaptations related to its pollination syndrome, resulting in an enormous variation in size, shape and surface structures^12,13^. In general, pollen distributed by wind are smaller, show less pronounced ornamentation and are usually spread as single grains, in contrast to those carried by insects^14^. In addition, pollen of angiosperms can be covered with a viscous pollenkitt layer^15^, which is also functional for the specific dispersal strategy, but usually reduced in anemophilous species. All these properties come along with further characteristics, like water content of grains, rate of water loss, pollen viability and distance of dispersal^16^ and they also affect the ability of pollen to adhere to a substrate or to each other. However, although several studies investigated the evolution of pollen grains, the selection mechanisms responsible for their current forms are not fully understood yet^17–19^. Furthermore, there is still not much known about the actual forces, acting on pollen grains during release, transport and capture.

Adhesion is a very important part of the pollination process. From a physical perspective, a pollen grain can be described as a micro particle on a surface. In general, at each stage of the pollination process, the pollen has to be kept in place until the conditions are optimal for transfer to the next place. For pollen release, the external forces need to exceed the adhesion forces and the other way round for pollen capture. Release forces can be generated actively by the plant in the form of catapulting mechanisms^20^, but in most species, pollen release occurs passively through aerodynamic or vibration forces^3^ or stronger adhesion to the surface of a pollinator. The main contributions to adhesion are due to Van der Waals forces, electrostatic forces and liquid bridge forces^21^, which all depend on the specific pollen grain structure and properties. Thus, comparing the adhesion properties of pollen from different species and analyzing, how they are affected by surrounding conditions, can help to answer the questions, why different shapes and ornamentations have been evolved and how do they contribute to the successful pollination.

Previous studies already addressed this topic using different methodological approaches. Atomic force microscopy (AFM) is an established method to precisely examine the surface-surface interaction of single micro particles and has been previously used to analyze the general adhesion properties of single pollen grains^22^ as well as effects of coating layers^23^ and humidity^24,25^ as well as the mechanisms that induce pollen binding to the female receptive organ (stigma)^26^. Another approach is the method of vibration, which has been used to investigate the forces necessary for pollen release from the anther in different plant species^11,27,28^. However, the interaction between pollen and stigma, as well as the release mechanisms of anthers, are highly species-specific and the surface properties of both, pollen grains and substrate affect the adhesion force. To separate these effects from each other and to enable comparable analysis of different species, a standardized method, using a universal, non-adhesive substrate is required. One study combined detailed AFM measurements on single pollen grains with a centrifugation experiment that allowed quantitative data analysis to investigate the effects of pollen aging over 7 days on glass as a standard substrate^29^, leading to the conclusion that aging of the pollenkitt can affect adhesion properties of pollen grains in insect pollinated plant species. Thus, wind pollinated plant species having pollen grains with less or no coating should show a different response to aging due to their reduced pollenkitt coating. As at least some pollen grains are known to be still viable after long distant transport over thousands of kilometers^2^, the analysis of such long-time effects also has some relevance.

Centrifugation in combination with microscopic observation is a simple and reliable method to investigate the reaction of microparticles to either compressive or tensile force and has been used for several decades, primarily in powder technology, for example to characterize the influence of particle diameter and plasticity^30,31^, as well as particle-substrate interactions^32,33^. With moderate efforts, it enables the analysis of hundreds of particles simultaneously on a freely chosen substrate, with more or less precise control over the applied force, depending on the instrumental setting. Depending on the plant species, this analysis can provide a large amount of quantitative data about attachment force, providing reliable differentiation between species and a foundation for discussions about functional aspects of species-specific characteristics of pollen grains. However, to the best of our knowledge, there were only a few studies that used a similar method for pollen grain analysis so far^29,34^ and they also only considered entomophilous plant species.

In this study, we tested the hypothesis that the adhesion properties of pollen from anemophilous species on glass as a standard substrate change over time in a different way than those of insect pollinated species^29^, due to differences in their surface coatings. Therefore, we analyzed pollen from four different anemophilous species in a mass centrifugation setup, as described in Huth et al. (2022)^29^. As primary specimens we chose the gymnosperm representative scots pine (*Pinus sylvestris* Linné) (Pinaceae), a grass species - perennial ryegrass (*Lolium perenne* Linné) (Poaceae) and a special case within the usually insect pollinated Asteraceae family - mugwort (*Artemisia vulgaris* Linné) (Asteraceae). These species were compared to data on ribwort plantain (*Plantago lanceolata*, Linné) (Plantaginaceae) as a member of the ambophilous genus *Plantago* that was collected in an equal but separate experiment. Furthermore, we compared these data to the results of previous analysis on the insect pollinated hairy catsear (*Hypochaeris radicata*, Linné) (Asteraceae)^29^. While *A. vulgaris* and *H. radicata* belong to the same plant family, all species are not close related and were chosen only due to their pollination ecology. The aim of these experiments was to quantify the amount of pollen grains that are detached from the glass substrate by a certain centrifugal force under different conditions and to provide hypotheses about the influence of certain properties on adhesion. In detail, we compared the combined effects of species-specific pollen grain properties, such as size, shape, surface microstructure and presence of surface coating, on the detachment force. Furthermore, we compared seven days old grains to fresh ones to examine the effect of aging in each species.

In addition, we made a more detailed analysis of *A. vulgaris* pollen, including a statistical comparison of six samples containing fresh pollen, examination of a longer period of aging, as well as long-term effects of constant centrifugation and no centrifugation. We also made weight measurements in order to calculate the mass of single *A. vulgaris* pollen grains and used SEM to characterize the size and shape of pollen from all species. Another aim of this study was to explore, whether this centrifugation technique can be established as a standard method for pollen adhesion analysis and which details need to be considered.

## Materials and methods

### Specimens

**Fig. 1:**
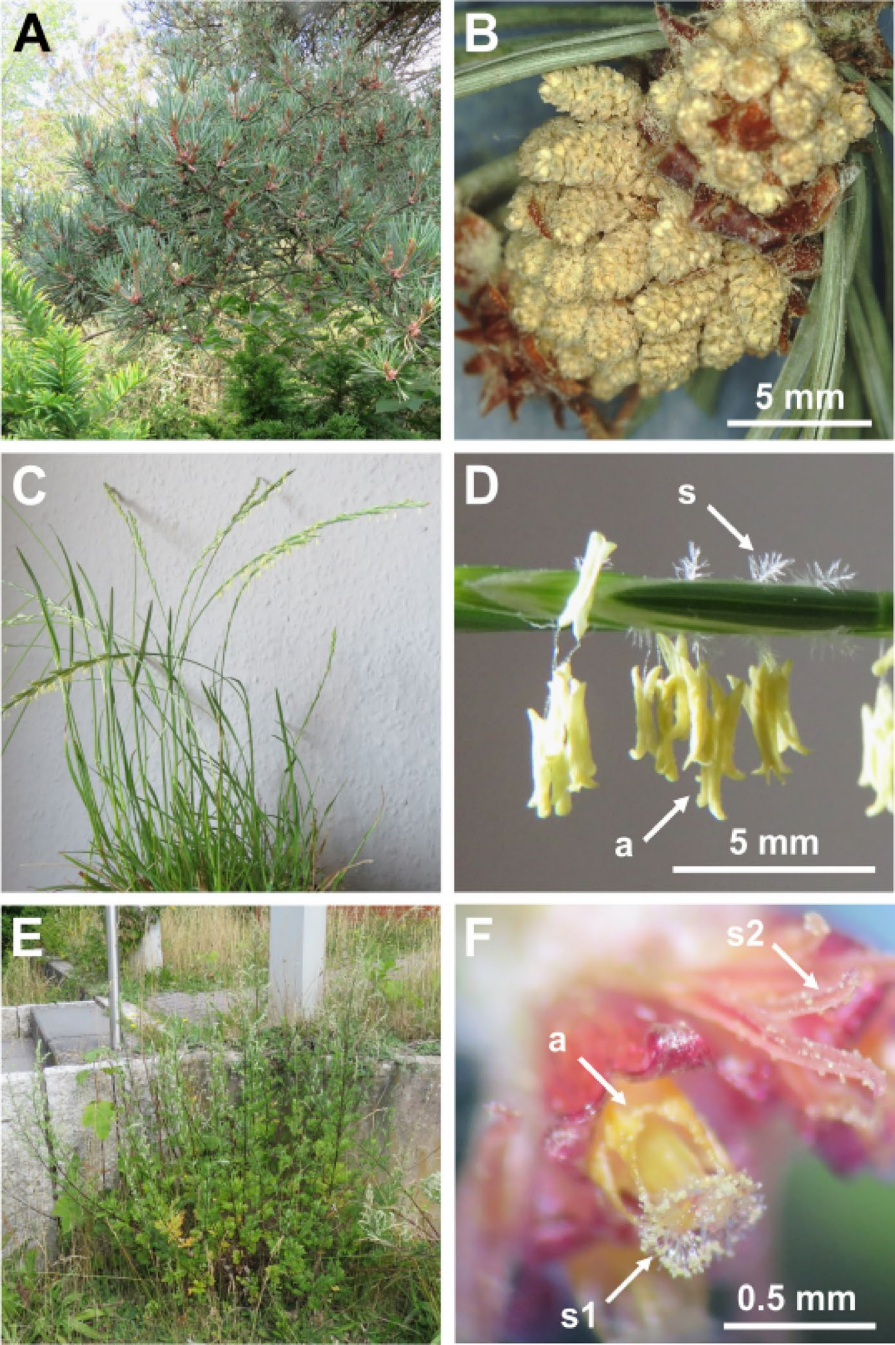
Images of plant species from our primary Experiment 1. (**A**) *Pinus sylvestris* close-up of spot where samples have been taken. (**B**) *P. sylvestris* male cones with pollen. (**C**) *Lolium perenne* indoor storage. (**D**) *L. perenne* close-up of flowers, arrows pointing at yellow open anthers (a) hanging down and white styles (s) standing upwards. (**E**) *Artemisia vulgaris*. (**F**) *A. vulgaris* close-up of fresh open flowers, arrows pointing at circular connected anthers (a) and unripe central style (s1) of a hermaphroditic flower and ripe style (s2) of a female flower.

**Fig. 2:**
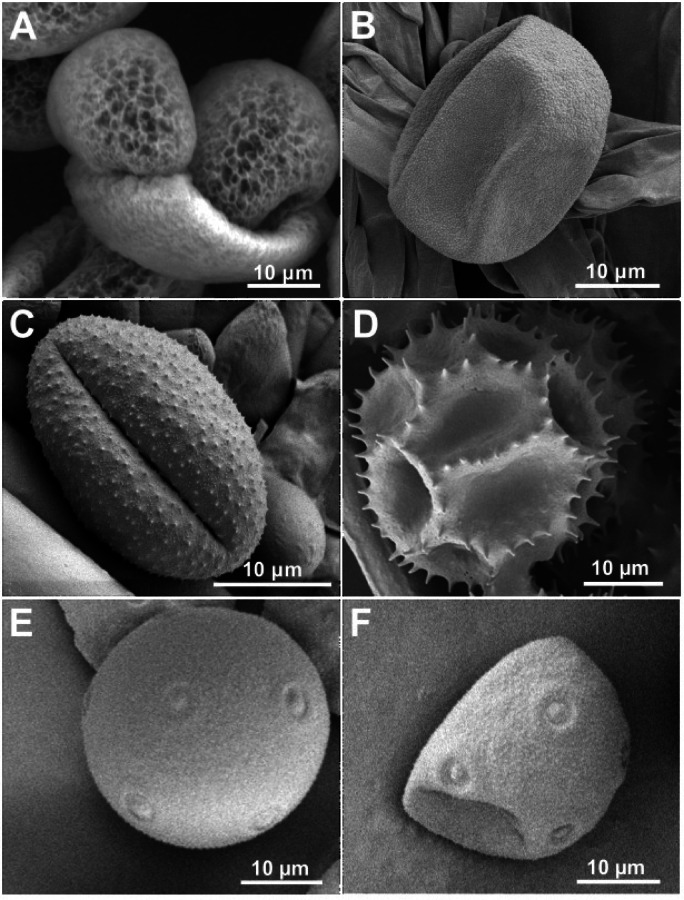
Scanning Electron Microscopy (SEM) images of pollen grains of all species studied. (**A**) *Pinus sylvestris*, (**B**) *Lolium perenne*, (**C**) *Artemisia vulgaris*, (**D**) *Hypochaeris radicata*, (**E**) *Plantago lanceolata* hydrated pollen. (**F**) *P. lanceolata* dehydrated crumpled pollen.

Pollen grains of *Artemisia vulgaris*,* Pinus sylvestris*, and *Lolium perenne* were collected from individual plants in the Botanical Garden of Christian-Albrechts-University Kiel, Federal country Schleswig-Holstein, Northern Germany. Samples of *P. sylvestris* were all taken from the same tree in a half shadow location in June 2023. For samples of *L. perenne*, a clump of grass of 10 cm diameter was taken in June 23 from a sunny spot at the beginning of its flowering period and kept indoor in a plant pod at room temperature (22°C) with regular daylight and watering. Samples of *A. vulgaris* were taken from individuals at four different sunny spots between July and September. Furthermore, samples of *P. lanceolata* were taken from green open spaces in the urban setting of Kiel during August and September and data from *H. radicata* were provided by Huth et al. (2022)^29^. All plant samples except for some *P. lanceolata* samples were taken from labeled individuals in the Botanical Garden of Kiel and were identified by the employees of the garden. Wild specimens of *P. lanceolata* were identified using “Rothmaler Exkursionsflora von Deutschland Gefäßpflanzen: Grundband, Editors: Eckehart J.Jäger, 21. editon (ISBN: 978-3-662-49707-4)” and by comparing them to labeled individuals. We did not deposit any plant material in a publicity available herbarium. Plant images are shown in Fig. 1, whereas SEM images of respective pollen grains are shown in Fig. 2.

### Centrifugation experiments

**Fig. 3:**
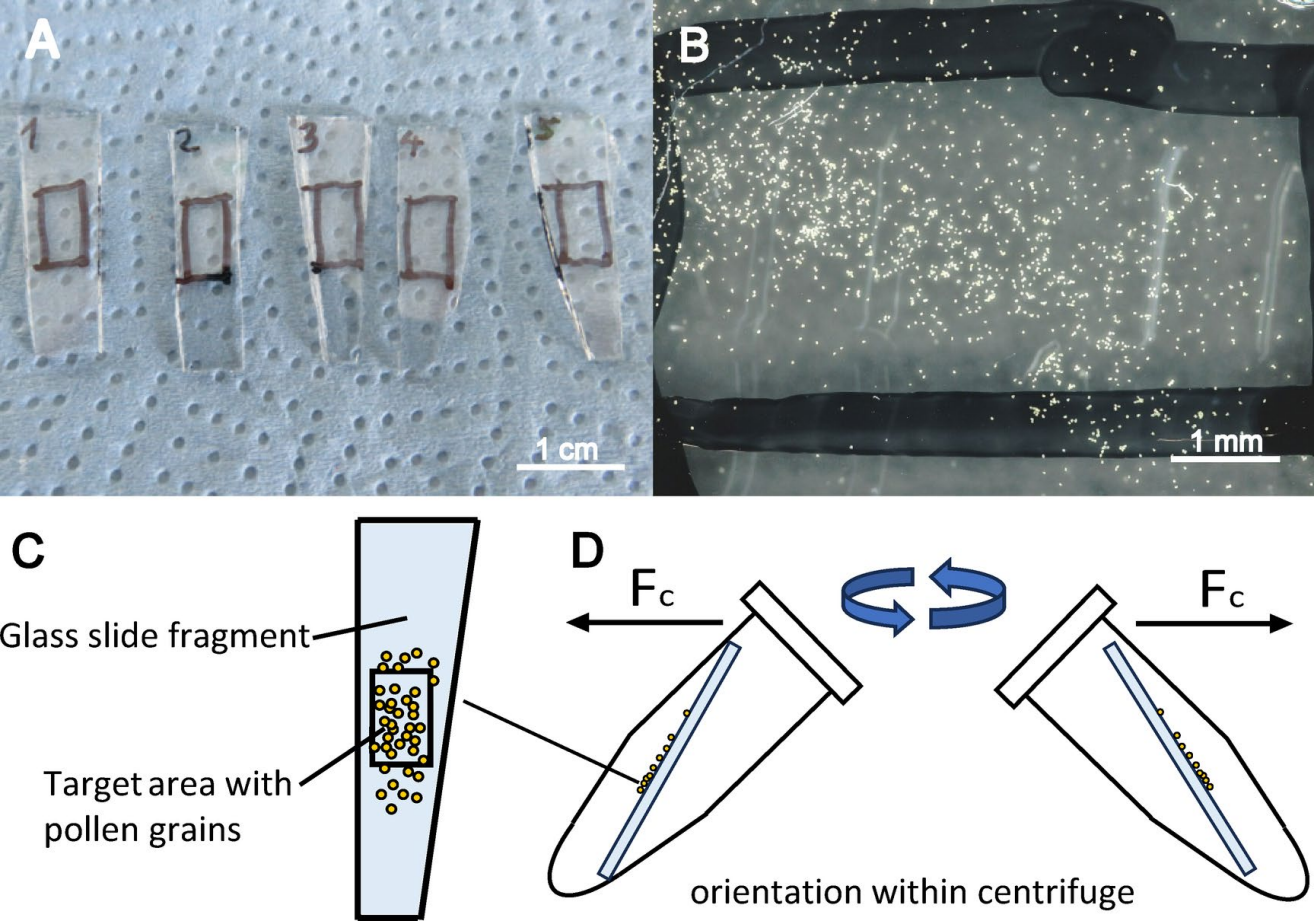
Centrifugation experiment. (**A**) Cleaned glass fragments with marked target area. (**B**) Image for analysis showing target area with pollen before centrifugation. (**C**) Scheme of glass fragment with target area. (**D**) Scheme of setup: pairwise positioned Eppendorf tubes with glass fragments within the centrifuge. Fc: Centrifugal force vector.

The experimental setup was previously described in Huth et al. (2022)^29^. The three species *P. sylvestris*, *L. perenne* and *A. vulgaris* were analyzed together in a first experiment (Experiment 1). The data on *P. lanceolata* has been collected in an equal experimental setup, but with a slight difference in centrifugation radius, forcing us to treat it as a separate experiment (Experiment 2) for evaluation, as described in the results section. As preparation, glass slides that served as the substrate for adhering pollen grains were cut into fragments, which fit well into a 2.5 ml Eppendorf tube. Each fragment was weighed and numbered, so they could be ordered pairwise for centrifugation. In the center of each fragment, a rectangular area of 3 × 8 mm was marked as the target area for pollen grains. A scheme of the setup is shown in Fig. 3. For sampling, inflorescences of each target species were cut off and pollen from randomly selected flowers were spilled on the target areas. For the species comparison experiment, we examined grains of each species in three different conditions (categories): “Fresh” pollen were spilled on glass immediately after collection and were immediately centrifugated. Pollen “in contact” were also immediately spilled on the target area, but stored for 7 days at room temperature and relative humidity of 50% before centrifugation. For “aged” pollen, the cut off inflorescences were kept in a plastic box at room temperature and relative humidity of 50% for 7 days, then the pollen were spilled on the glass and immediately centrifugated. For more detailed analysis of *A. vulgaris*, the categories “aged” and “in contact” were further subdivided into “2 h”, “1 day” and “7 days”.

For each species and category, several sets of pollen were centrifugated at once, as simultaneous replications of each setting. Before pollen spilling, all glass fragments were kept in closed glass Petri dishes and cleaned with 70% ethanol. The glass fragments carrying pollen were placed within Eppendorf tubes (see Fig. 3) and centrifugated for 3 min with a Heraeus Sepatech Biofuge A table centrifuge (Heraeus Holding GmbH, Hanau, Germany) in a series of increasing rotation (1000 rpm, 2000 rpm, 3000 rpm, … to a maximum of 10000 rpm).

Before and after each centrifugation, the fragments were taken out of the tubes to photograph the target area with a Keyence 3D measurement macroscope (Keyence Deutschland GmbH, Neu-Isenburg, Germany), at 40 x magnification, resulting in a series of 11 images for each sample. From these images, the number of single pollen grains that remained attached to the target area after each rotation speed was counted.

For a more detailed analysis on *A. vulgaris*, we added two more settings. For the setting “constant rotation”, 4 samples were centrifugated 10 times for 3 min with only 1000 rpm, to test, whether the attachment properties change in response to the constant centrifugal force, due to short-term pollen aging. In addition, 4 samples for the setting “no rotation” were treated exactly the same way as all other samples, but without putting them into the centrifuge. This was done as a negative control to test how many grains get detached by just handling the samples. This experiment was repeated after 1 day, 3 days and again after 7 days, always with the same 8 samples. However, the counting software failed to evaluate the results after 3 days of the experiment, due to different light conditions in the images. Therefore, we excluded all rounds of that day, resulting in a final series of 30 rounds, referring to three states of age (“day 1”, “day 2” and “day 8”).

### Weight measurements of *A. vulgaris* pollen

To determine the weight of single *A. vulgaris* pollen, we made a series of 10 weight measurements with an UMX2 Ultra-microbalance (Mettler Toledo, Columbus, USA), with each weight-sample containing several hundred to thousands of pollen grains. Before each measurement, the balance pan was cleaned with 98% ethanol and the empty dry pan was used to tare the balance. The exact number of grains per sample was counted manually by marking the grains in Inkscape software (version 1.3, Inkscape-Project) on images of the samples, taken before each measurement. In some cases, the number of grains in a clump needed to be estimated. From these results, the average mass of a single grain was calculated by plotting the measured weight [ng] against the number of grains and analyzing the trend line. This procedure was done twice, for fresh pollen, as well as for 7 days old grains. Both measurement series were compared statistically, using Sigma Plot (version 12.5, Systat Software GmbH, Frankfurt am Main, Germany).

### Data evaluation

The basis for our analysis is the assumption that in the moment when a pollen grains gets detached from the substrate, its maximal adhesion force (***F***_***A***_) is reached, which is equal to the applied centrifugal force (***F***_***c***_). The actual centrifugal acceleration ***a***_***c***_ [m/s^2^] acting on each pollen grain can be described with the term

$$\it \:\text{a}_{c}\:=\:4{\varvec{\pi\:}}^{2}\times\:\:\text{r}\:\times\:\:{\varvec{n}}^{2}$$

with ***r*** as the distance between pollen grains and the center of the centrifuge [m] and ***n*** as the rotation in [rev^− 1^]. Adding the mass ***m*** of a single pollen grain [µg], the actual centrifugal force ***F***_***c***_ [nN], acting on each pollen grain, can be described with the term

$$\it \:{\varvec{F}_{c}\:=\:4\:\varvec{\pi\:}}^{2}\:\times\:\:\varvec{r}\:\times\:\:{\varvec{n}}^{2}\times\:\varvec{m}\:=\:\varvec{F}_{A}$$

As the pollen grain mass was not measured for all species, we decided to use the safety factor (**SF**) which is the force per weight, as basis for comparison. Adding the weight force of a single pollen grain (***F***_***g***_ = ***m*** × ***g*** with ***g*** = 9.81 m/s^2^), the safety factor is given by

$$F_{c} /F_{g} = (a_{c} \times m){\text{ }}/{\text{ }}(m \times g){\text{ }} = a_{c} /g$$

In this case, the safety factor is equivalent to the relative centrifugal force (**RCF =** ***a***_***c***_ ***/ g***), which is a dimensionless value, used to compare different centrifugal accelerations as multiples of gravity.

The radius ***r*** was determined geometrically, considering the distance between the rotation axis and the upper end of the tube, the depth of the target area within each tube and the maximal angle of tubes within the centrifuge.

Pollen grains within the target area were counted from the images using the particle analysis tool of ImageJ (version 1.54d, Wayne Rasband and contributors– National Institutes of Health, Bethesda (Maryland), USA). For this purpose, we determined the size range of single grains (projected particle area) in px^2^ with a circularity of 0.7-1.0 and adjusted the threshold of brightness to exclude all other structures from counting. Pollen clumps were excluded as well, only single grains were counted. Statistical analysis was done in Sigma Plot (version 12.5, Systat Software GmbH, Frankfurt am Main, Germany).

## Results

### Comparison of species

In total, during Experiment 1 we centrifugated 6 samples of *A. vulgaris*, 4 samples of *L. perenne* and 4 samples of *P. sylvestris*, referring to the category “fresh”. For the category “aged”, we analyzed 2 *A. vulgaris* samples, 4 *L. perenne* samples, 4 *P. sylvestris* samples. Finally, the category “in contact” contained 2 *A. vulgaris* samples, 6 *L. perenne* samples and 4 *P. sylvestris* samples. Experiment 2 on *P. lanceolata* consisted of 4 samples per category, except for category “in contact”, which only consisted of 3 samples. In addition, we made a new evaluation of *H. radicata* data from Huth et al. (2022)^29^, which provided results for 4 samples for each category. For each set of sample-replications, mean value and standard deviation were calculated and used as basic values for comparison.

At high rotation speed (8000 rpm or more), some glass fragments crushed inside the Eppendorf tubes, which forced us to stop counting. Nevertheless, we counted pollen for up to 10,000 rpm for almost all settings. Only for “fresh” *L. perenne* pollen, we got no values for the last round (10000 rpm), so we kept the 9000 rpm values as the final ones, as no more rotation speeds could be tested. The total number of grains per setting strongly varied from 149 ± 74 to 678 ± 179 (*A. vulgaris* “in contact” and “aged”). Therefore, we compared the percentage of detached grains referring to the initial number, which is equivalent to the detachment probability. Furthermore, we chose round 1, 2 and 3 (1000–3000 rpm) in detail as characteristics for comparison, as well as the sum of grains detached in round 4–10 and the number of remaining grains after round 10.

**Fig. 4:**
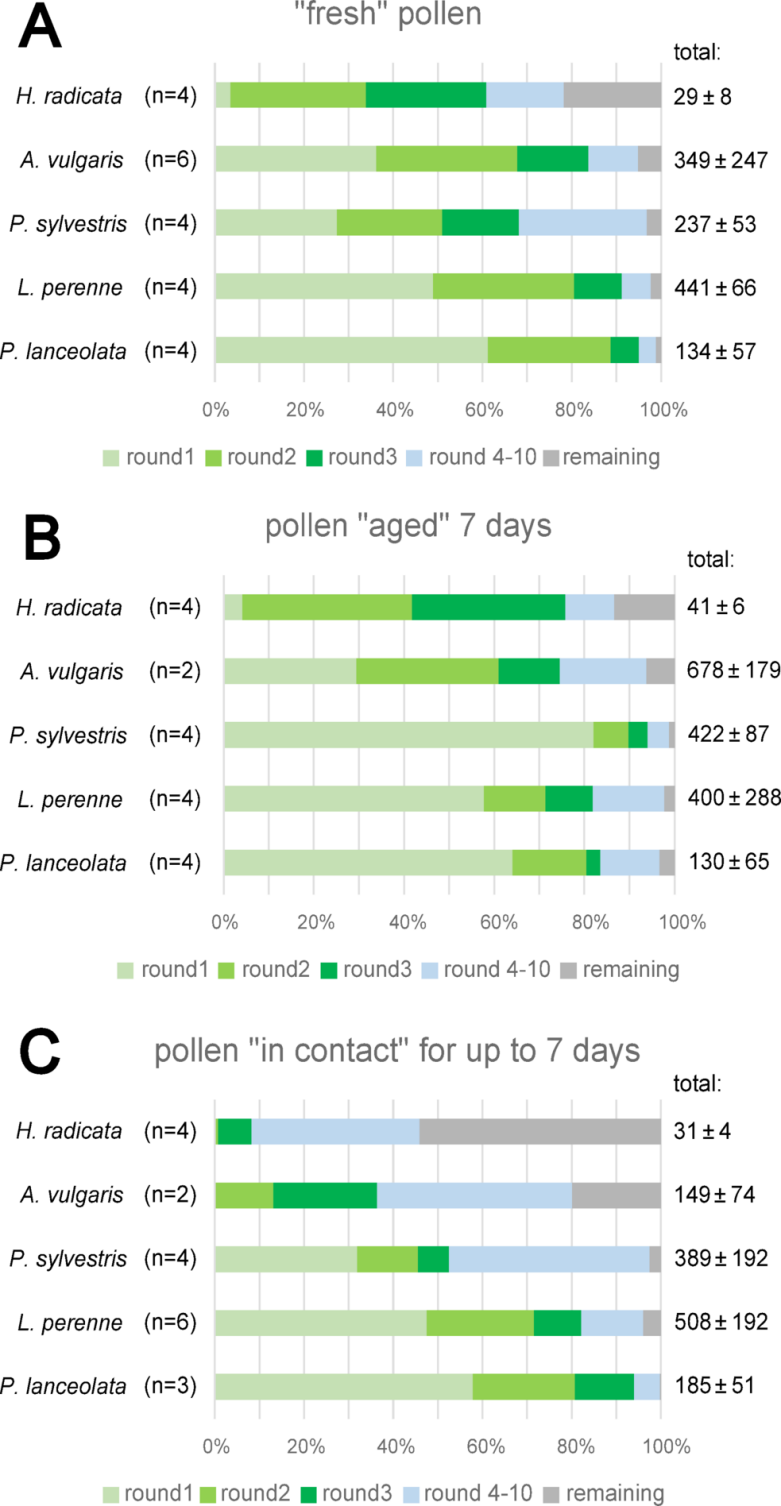
Summary of adhesion performance of pollen grains for all species and categories, showing the percentage of detached grains per round, which represent the distribution of measured adhesion safety factors (SF) in % for each category. (**A**) “Fresh” pollen. (**B**) “Aged” pollen. (**C**) Pollen “in contact”. Corresponding safety factors are listed in Table 1. “Total” values represent the number of grains per category and species tested as mean and standard deviation, while “n” shows the number of replications.

The geometric determination of the centrifugal radius resulted in a value of 0.066 m. Due to the fact that the handcrafted glass fragments slightly varied in their shape, this value comes with an uncertainty of 3%, resulting in a radius of 0.066 ± 0.00189 m. A similar error can also be assumed for the angle of glass fragments within the Eppendorf tubes, which results in an angle of 51.0° ± 1.5° between the glass substrate surface and centrifugal force vector during rotation. As a consequence, the centrifugal force and the safety factor must be treated with the same uncertainty. The three species examined in Experiment 1 (*A. vulgaris*, *L. perenne* and *P. sylvestris*) were characterized by a safety factor **SF1**, reaching from 73.779 to 7377.890. The samples of *P. lanceolata* had been centrifugated with a slightly different radius of 0.060 m, due to the experimental setting. For *H. radicata*^29^ our recalculation also provided a radius of 0.060 m (own observation). Therefore, these results were included in Experiment 2 and both species were characterized by a lower safety factor **SF2**, reaching from 67.418 to 6741.826. Detailed values of safety factors are listed in Table 1. These values represent the maximal measured adhesion safety factor of all pollen grains that got detached in the corresponding round. The averaged detachment distribution across the increasing safety factor for all species and categories is summarized in Fig. 4. A detailed evaluation of Fig. 4 is provided in the Supplemental Materials.

**Table 1:** Summary of calculated safety factors from experiment 1 and 2, referring to specific measurement rounds (see Fig. 4).

Comparison of all species - corresponding safety factors		
	Experiment 1	Experiment 2
Species	*A. vulgaris*	*P. lanceolata*
	*L. perenne*	*H. radicata*
	*P. sylvestris*	

	Safety factors (SF 1)	Safety factors (SF 2)
Round 1	< 73.779	< 67.418
Round 2	< 295.116	< 269.673
Round 3	< 664.010	< 606.764
Round 4–10	664.010–7377.890	606.764–6741.826
Remaining	> 7377.890	> 6741.826

### “Fresh” pollen

**Fig. 5:**
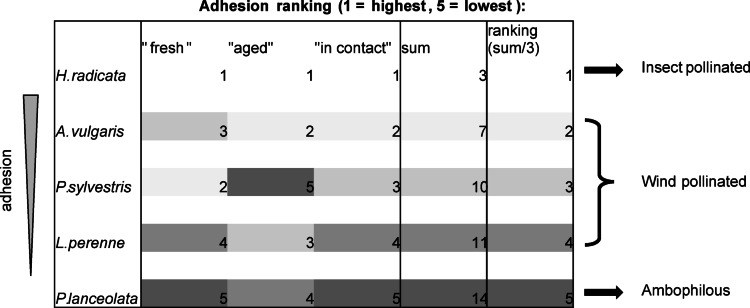
Adhesion ranking for all samples and categories, based on the distribution of detached grains over 10 rounds shown in Fig. 4. The percentage of grains per round was multiplied with the corresponding safety factor (SF) (see Table 1) and added up to calculate the total adhesion for each sample, ordering the species from highest adhesion (1) to lowest adhesion (5) in sum. (Values represent relative amount of adhesion in relation to the other species). The mean values over all three categories for each species result in the final ranking. The corresponding pollination ecology for each species is shown on the right-hand side.

Among all species, *H. radicata* has the highest maximal safety factor (MSF) for fresh pollen, referring to the majority of grains, with a value of 223.6 and *P. lanceolata* has the lowest MSF of 67.4. The majority of grains of the three remaining species detached in the first round with a slightly higher MSF of 73.8, which corresponds to a G-force of 74. However, this result was most pronounced for *L. perenne* and fewest for *P. sylvestris*. Also in general, over the whole range of increasing SF, pollen of *H. radicata* showed the best adhesion, followed by *P. sylvestris* and *A. vulgaris*, while the SF distribution in *L. perenne* and *P. lanceolata* was obviously shifted to lower SFs. The overall adhesion ranking for all species and categories is summarized in Fig. 5.

### “Aged” pollen

For the category “aged”, pollen of the two species *H. radicata* and *P. sylvestris* tended to lower adhesion and increasing detachment during the first three rounds in comparison to “fresh” pollen. Referring to the distribution across the safety factors, it can be concluded that the three species *P. lanceolata*,* A. vulgaris* and *L. perenne* slightly shifted their SFs to higher values and stronger adhesion, while *H radicata* slightly and *P. sylvestris* dramatically shifted them to lower adhesion. However, the absolute values of MSF for the majority of grains remained the same as for “fresh” pollen, except for *A. vulgaris*, as for this species the majority of “aged” grains detached with a higher MSF of 295.1 in the round 2.

### Pollen “in contact”

The category “in contact” showed the strongest difference overall, compared to both “fresh” and “aged” pollen and this was more or less pronounced for each species. For *P. lanceolata*, the distribution of grains “in contact” was more similar to “fresh” pollen than to “aged” one, as 94.0% of them detached during the first three rounds. In *P. sylvestris* and *L. perenne* all three categories were equally different from each other, while this difference was much stronger in *P. sylvestris*. In detail, only 52.5% of *P. sylvestris* grains detached during the first three rounds, which was less than for “fresh” pollen and much less than for “aged” one. For *L. perenne*, more than 80% of grains detached in rounds 1–3, but with some differences in each round. *H. radicata* showed the strongest difference with only 8.2% loss in the first three rounds, directly followed by *A. vulgaris* with 37.2%. For both these species, this was much less detachment than for “fresh” and “aged” pollen. Also, for both these species, there was no detachment at all in the round 1 and the strongest detachment shifted to the round 3 with 7.4% in *H. radicata* and 23.8% in *A. vulgaris*.

For the safety factors, one can conclude that for *L. perenne* and *P. lanceolata* there was only a slight difference in comparison to the other categories. For *P. lanceolata*, pollen “in contact” showed even lesser attachment than “aged” ones. However, for the other three species, adhesion strongly increased. Almost 50% of *P. sylvestris* and more than 60% of *A. vulgaris* pollen had safety factors of more than 664.0, which corresponds to a G-force of 664. For *H. radicata*, over 90% of pollen had SFs of more than 606.8 and more than 50% had SFs beyond the maximal centrifugal acceleration (> 6741.8). Also 20% of *A vulgaris* pollen had a safety factor of more than 7377.9, marking grains of these two species as the most adhesive ones overall, followed by *P. sylvestris*,* L. perenne* and finally *P. lanceolata* as the least adhesive one (see Figs. 4 and 5).

### Weight measurements in *A. vulgaris* and force calculation

**Fig. 6:**
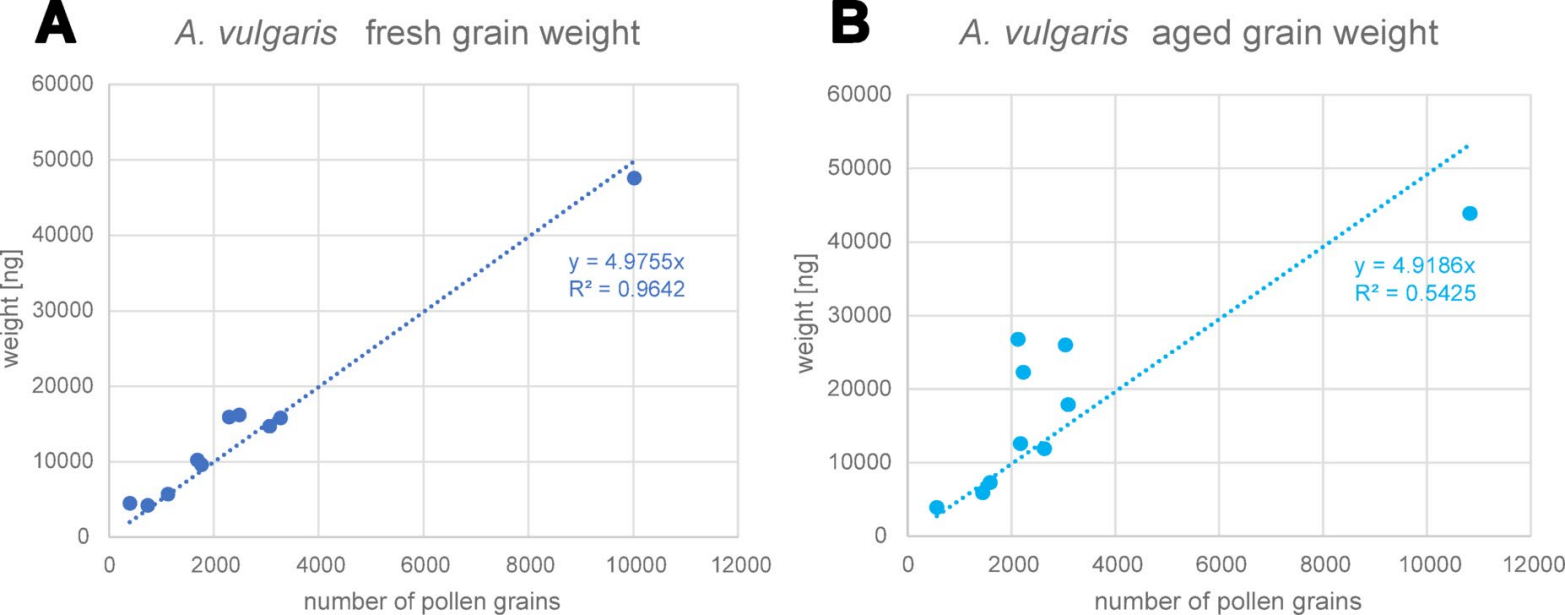
Summary of weight measurements for *A. vulgaris* pollen, showing the measured weight [ng] referring to the counted number of grains. (**A**) Results for fresh grains, weighted immediately after collecting them from the plant. (**B**) Results for aged grains, weighted 7 days after collection. Number of samples per setting was 10. The simple linear regression (SLR) based on ordinary least square method (OLS) provides the corresponding mass of a single pollen grain and the certainty of the measurements (R^2^).

In the samples collected for weight measurements, the number of counted pollen grains per sample reached from 398 to 3274 for 9 of 10 “fresh” probes, with an additional extreme value of 10,020 grains in the tenth sample. For 7 days old grains, the values ranged from 561 to 3089, also with one additional extreme value of 10,829 grains. The calculation of pollen mass from all 10 “fresh” samples provided a proportional correlation of weight [ng] to the number of grains = 4.9755 with a coefficient of determination of R^2^ = 0.9642. This results in the value of 4.976 ng for the average mass of a single “fresh” pollen grain of *A. vulgaris*. In the same way, the analysis of 7 days old grains provided a slightly lower average mass of 4.919 ng for a single “aged” or “in contact” pollen grain. However, this value came with a higher uncertainty due to a lower coefficient of determination of R^2^ = 0.5425. As normality test failed, we used a non-parametric test for statistical comparison between fresh and 7 days old samples. As a result, the test showed that there is no significant difference in the pollen mass between “fresh” and “aged” pollen of *A. vulgaris* (Mann-Whitney-U test, n_1,2_=10, U = 49, t = 106, *p* = 0.970, alpha = 0.05). Nevertheless, we used the exact measured mass for further analysis, resulting in slightly different values for the centrifugal force. As a consequence, the centrifugal force reached from 3.601 nN (1000 rpm) to 360.076 nN (10000 rpm) for “fresh” pollen and from 3.560 nN to 355.951 nN for “aged” and “in contact”. The latter force range was also assumed for the subdividing categories “2 h” and “1 day”, despite pollen of that age have not been weighted directly. Thus, these values represent the measured range of adhesion force for *A. vulgaris* pollen grains. Force values are summarized in Table 2. A summary of weight measurements is shown in Fig. 6.

**Table 2:** Summary of calculated maximal adhesion force for fresh and dry *A. vulgaris* pollen grains, referring to specific measurement rounds (see Fig. 7).

Artemisia vulgaris detailed analysis – maximal adhesion force		
Categories	Fresh weight	Aged weight
	4.976 ng	4.919 ng

	Force [nN]	Force [nN]
Round 1	< 3.600	< 3.559
Round 2	< 14.403	< 14.238
Round 3	< 32.407	< 32.035
Round 4–10	57.612–360.076	56.952–355.951
Remaining	> 360.076	> 355.951

### Statistical analysis of “fresh” *A. vulgaris* pollen

In addition to considering the mean value for comparison of species, the six samples containing “fresh” *A. vulgaris* pollen were compared statistically by counting the measured maximal adhesion force and the maximal adhesion SFs respectively of every single pollen grain as single values and therefore considering each sample separately. Following this approach, the sample 1 had the highest total sample size of 744 pollen grains (*n* = 744). Furthermore, we got the sample 2 with *n* = 564, the sample 3 with *n* = 299, the sample 4 with the lowest sample size of *n* = 98, the sample 5 with *n* = 202 and the sample 6 with *n* = 200.

**Fig. 7:**
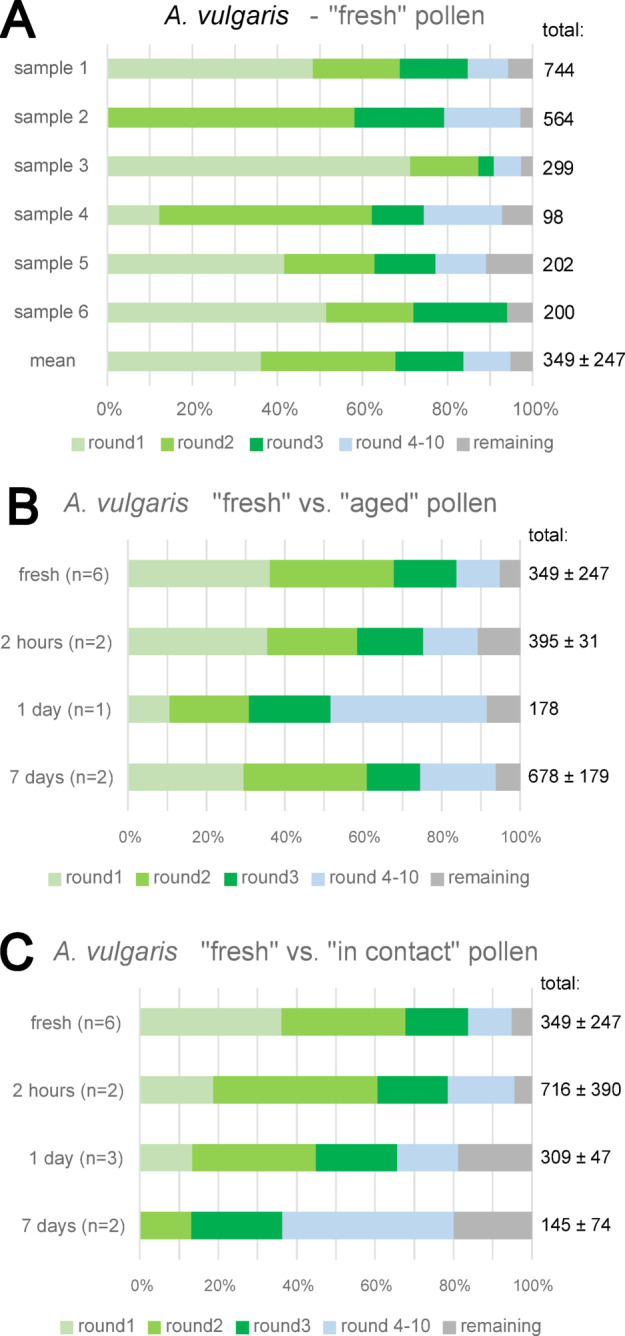
Summary of adhesion performance of pollen grains from detailed experimental analysis on *A. vulgaris*, showing the percentage of detached grains per round, which represent the distribution of measured adhesion forces and safety factors in % for every setting. **A**: Summary for “fresh” pollen. **B**: Summary for “aged” pollen. **C**: Summary for pollen “in contact”. Corresponding adhesion forces are listed in Table 2. “Total” values represent whole number of grains per setting as mean and standard deviation, while “n” shows the number of replications.

In detail, the distribution of detachment over 10 rounds, as described above for all species, showed strong variation among the single *A. vulgaris* samples, ranging from 0 to 70% in the round 1, from 16 to 60% in the round 2 and 4–22% in the round 3. Nevertheless, the great majority of grains in all samples detached during the first three rounds, as only 0–18% detached during rounds 4–10 and 3–7% in total remained after the round 10. The results are summarized in Fig. 7A.

For statistical analysis, we considered only the detached grains, as no actual force was measured for the remaining ones. As a consequence, the sample size slightly decreased for the sample 1 to 701 measured force values (*n* = 701). The size of samples 2 (*n* = 565), 3 (*n* = 303), 4 (*n* = 94), 5 (*n* = 184) and 6 (*n* = 196) also slightly decreased. As normality test failed, we used non-parametric tests for comparison. Kruskal-Wallis- One-Way-ANOVA on ranks revealed a significant difference between all six samples with a p-value < 0.001 (DF = 5; H = 289.013; alpha = 0.05). The same result was found for the percentage of grains instead of the total counting (ANOVA on ranks, n_1 − 6_ = 90–100; DF = 5: H = 98.815, *p* < 0.001, alpha = 0.05).

In addition, pairwise comparison, using Mann-Whitney-U-Rank-Sum-test, showed significant difference in almost any combination. Only between samples 1 and 5, as well as between samples 5 and 6, there was no significant difference (*p* > 0.05). Considering the percentage, this result was less pronounced, as in two remaining cases, between samples 1 and 6 and between samples 2 and 4: the difference was not significant (*p* > 0.05).

### Measurements over time in *A. vulgaris*

The detailed measurements of *A. vulgaris* over time are summarized in Fig. 7B, C. These measurements revealed some further differences due to pollen age. It can be said that the general adhesion force of “aged” *A vulgaris* pollen increased during the first 24 h, as almost 50% of “1 day” old grains got detached by a force of more than 57.0 nN, compared to only 16% of “fresh” pollen. Already after “2 h”, this percentage increased to nearly 25%, but after “7 days” it decreased again to 26%. The highest adhesion showed almost 11% of “2 h” old remaining grains that even resisted the maximal centrifugal force of 356.0 nN, which corresponds to a G-force of 7378.

The general adhesion force for pollen “in contact” constantly increased over time and was strongest for “7 days” old pollen, as more than 60% had an adhesion force stronger than 57.0 nN, compared to 34% of “1 day” old pollen, 21% of “2 h” old pollen and 16% of “fresh” pollen. In addition, about 20% of “1 day” old grains and “7 days” old grains, respectively, resisted the maximal centrifugal force of 356.0 nN. Therefore, we can conclude that pollen of *A. vulgaris*, which aged in contact for more than 1 day, show the strongest increase of adhesion. A detailed evaluation of Fig. 7 is provided in the Supplemental Materials.

### Long time experiments in *A. vulgaris*

**Fig. 8:**
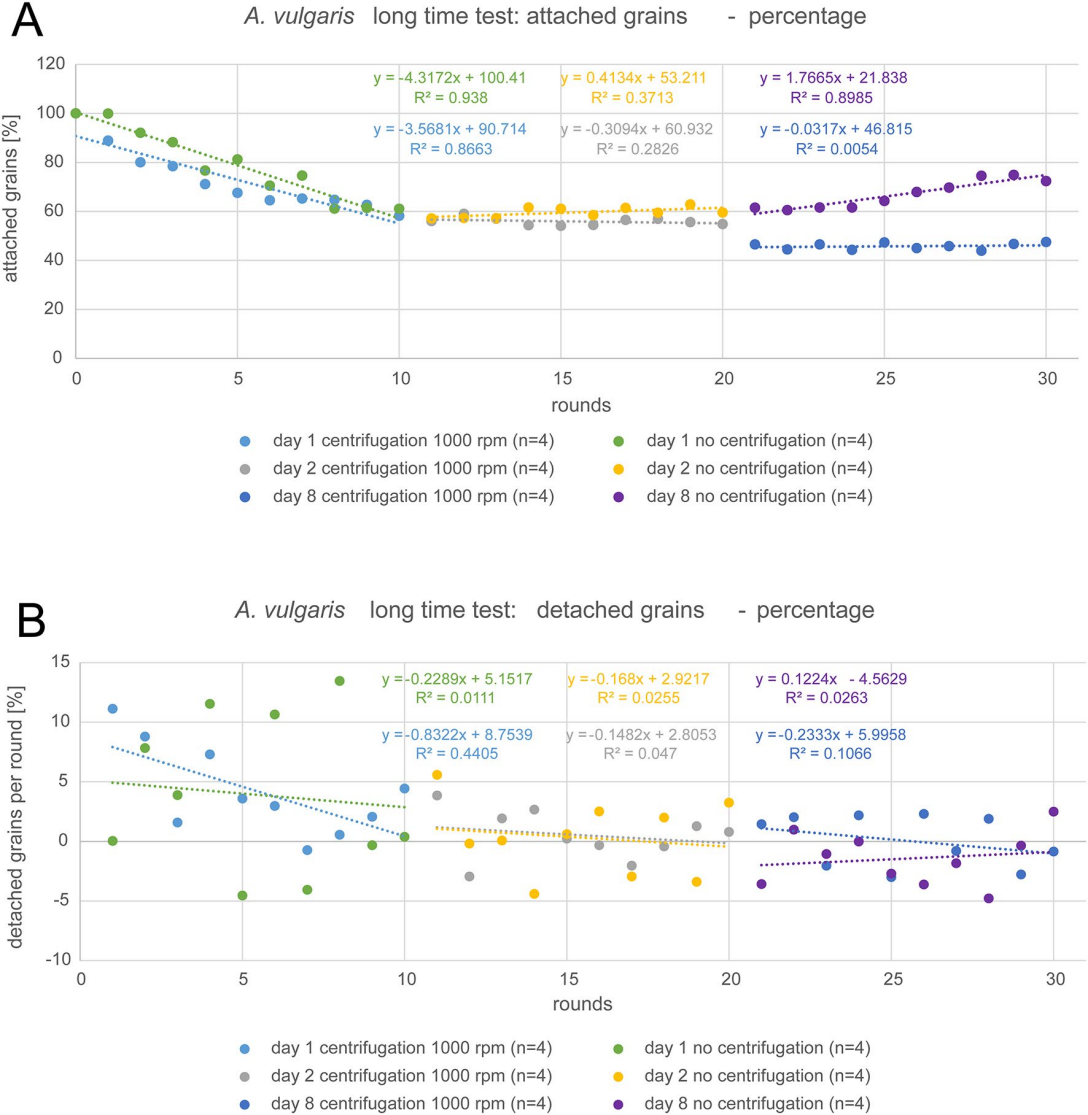
Summary of long time analysis of *A. vulgaris* pollen grain adhesion for the experimental settings “constant rotation” with 1000 rpm for 3 min and “no rotation” as control (n = replications per setting). (**A**) Percentage of attached grains per round with corresponding simple linear regression lines. (**B**) Percentage of detached grains per round with corresponding simple linear regression lines (SLR) based on ordinary least square method (OLS).

The long time analyses of “constant rotation” (1000 rpm) and “no rotation” gave some insights into further changes of pollen adhesion behavior. We considered the overall detachment for each day and also used simple linear regression to characterize the tendency over 10 rounds and its coefficient of determination. During the first day, about 40% of pollen from all samples detached. Interestingly, this was rather similar for both categories, although for “constant rotation” more grains detached during the first rounds, while detachment in “no rotation” samples was more random. For “day 2” samples, there was almost no detachment. For “constant rotation”, the number of counted grains only decreased from 56 to 55% and “no rotation” samples even showed a slight increase from 57 to 60%. This was probably due to the presence of dust particles, which could attach to the glass fragments, despite closed storage. This effect was far more pronounced on “day 8”, as the number of counted particles in “no rotation” samples strongly increased from 62 to 72%. While visual observation clearly showed the contamination, the counting software was unfortunately not able to distinguish between pollen grains and dust particles of a similar size and shape. On the other hand, the number of grains in “constant rotation” experiment decreased between “day 2” and “day 8” from 55 to 47%, but showed no further decrease during the last series of centrifugation. The results are shown in Fig. 8A.

The number of detached grains in each round (Fig. 8B) provided some further insights. While grains of all samples constantly detached during the first day, the number of detached grains per round fluctuated around a value of 5%, while the trendline indicated a decrease of detachment over all 10 rounds, which also corresponds to the observations on samples centrifugated with an increasing force. This trend was present for both categories, but more pronounced in the “constant rotation” experiment (R^2^ = 0.4405) than the “no rotation” one (R^2^ = 0.0111). Detachment per round during “day 2” also showed fluctuation, but around a lower value of nearly 0% with slight trends of decrease, which was almost identical for “constant rotation” (R^2^ = 0.0470) and “no rotaion” (R^2^ = 0.0255) cases. However, for “constant rotation”, this decrease was less pronounced than during “day 1”. During “day 8”, the detachment per round in “no rotation” cases slightly increased again (R^2^ = 0.0263), but fluctuated around a value below 0%. The loss of grains was obviously not enough to compensate the increasing contamination with dust particles. For “constant rotation”, there was still a decrease with fluctuation around 0%, which was similar to “day 2”, but with slightly less uncertainty (R^2^ = 0.1066).

**Fig. 9:**
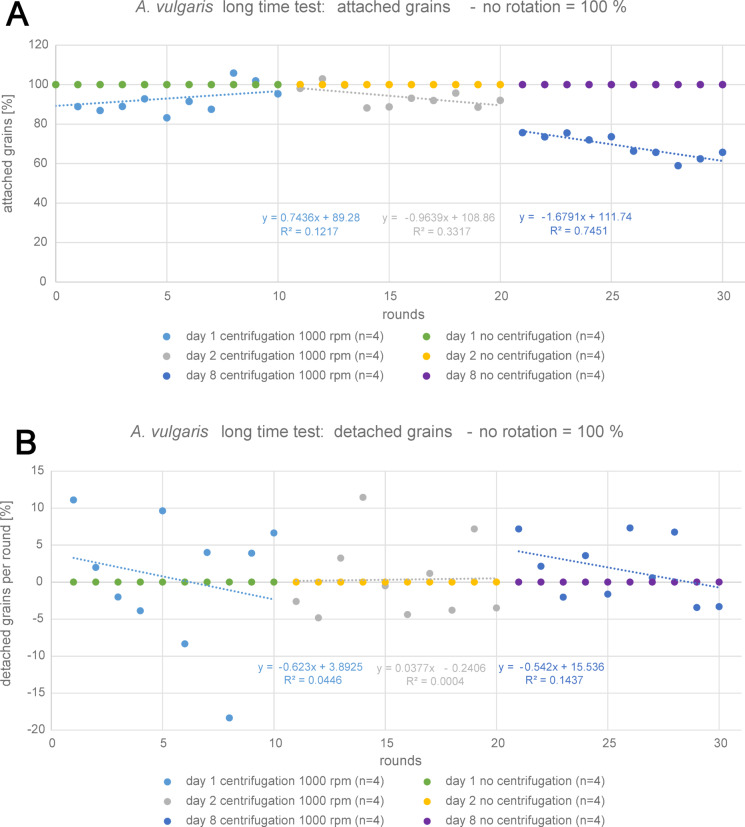
Summary of long time analysis of *A. vulgaris* pollen grain adhesion for the experimental setting “constant rotation” with 1000 rpm for 3 min, as percentage of the “no rotation” control setting (n = replications per setting). (**A**) Percentage of attached grains per round with corresponding simple linear regression lines. (**B**) Percentage of detached grains per round with simple corresponding linear regression lines (SLR) based on ordinary least square method (OLS).

In a second analysis, we defined the total number of attached grains from “no rotation” experiments in each round as 100% and each value of “constant rotation” was given as percentage of that value for every round, respectively (Fig. 9). With this method, we excluded the effect of random loss that was caused by sample handling on the “constant rotation” category, in order to isolate the actual true effect of the centrifugal force from the systematic error caused by the sample manipulation. However, in this context, the results of “day 8” must be treated with caution, due to the dust contamination of samples in “no rotation” experiment.

Referring to this analysis, the centrifugal force had no additional effect on detachment during “day 1”, as the number of attached grains in reference to the “no rotation” control even slightly increased (R^2^ = 0.1217). On the other hand, during “day 2” and “day 8” there was additional detachment, which exceeded the “no rotation” loss about 10% at “day 2” (R^2^ = 0.3317) and about 18% at “day 8” (R^2^ = 0.7451). Also referring to the number of detached grains per round, the results again showed a tendency of decrease for “day 1” (R^2^ = 0.0446) and “day 8” (R^2^ = 0.1437), which means that the effect of the centrifugal force itself on detachment decreased over 10 rounds. However, for “day 2”, there was no tendency due to the too strong scattering of the values (R^2^ = 0.0004). In all cases, fluctuation was around 0%. Thus, we may conclude that the effects of constant rotation and sample handling (no rotation) indicate an average methodical error of 0 to 5% for this study.

## Discussion

### Sampling and Preparation

The experimental setup, used in this study, proved to be a very effective method to study the adhesion properties of high numbers of pollen grains. With a record value of 991 *A. vulgaris* pollen grains, counted in one target area from the first image, even a single sample provides a great amount of quantitative data. However, effects of local or individual conditions, referring to the plant and its micro-habitat might affect the results, depending on the sampling strategy. For example, previous studies on *P. lanceolata* showed that populations of this species differ in their amount of pollen clumping, possibly due to the different amount of pollenkitt and that this difference correlates with their local habitat^35^. In this context, the number of replications, the amount of examined individuals and the overall randomization between samples need to be very high to collect truly representative data for the species. Nevertheless, even results from one individual can provide insights into species specific adaptations and enable comparison to other species.

### Influence of centrifugation parameters

The effect of the centrifugation radius was clearly shown by comparing our results to those previously provided by Huth et al. (2022)^29^. Therefore, we calculated the single grain mass of *H. radicata* pollen by dividing the centrifugal forces^29^ by our measured centrifugal acceleration. This resulted in a single grain mass of 7.619 ng for *H. radicata*, which was about 30% less than the mass found in previous studies. With the same method, we found a similar discrepancy for the data about *P. lanceolata*, resulting in a pollen grain mass of 9.355 ng. For *H. radicata*, Huth et al. (2022) determined a pollen mass of 10.86 ng (not published yet) and previous measurements on *P. lanceolata* delivered a pollen mass of 14.40 ng^27^. Although we can not exclude the possibility that the pollen mass shows strong variation due to changes in water content^16^, the similarity of both discrepancies indicates a methodical error.

In general, centrifugal force calculation is rather unsusceptible to methodical errors, as there are only two parameters, such as rotation speed and radius and both can be measured or set with high certainty, leaving the mass of the sample itself as the only critical value. As the rotation speed used in Huth et al. (2022)^29^ was exactly the same as in our study, the discrepancy can only correlate with the rotation radius. Finally, calculation of the centrifugal acceleration by dividing the forces measured in Huth et al. (2022)^29^ by the mass of individual *H. radicata* pollen grains (10.86 ng) resulted in an exact value for the used radius of 0.049 m. Our own observations showed that this value is unrealistic for the used centrifuge. We were able to detect an error during automatic calculation of radius that systematically leads to an underestimation of 20%. Therefore, we provide a corrected value for the radius of 0.060 m and also a correction of + 20% for the force and SF values of *H. radicata*^29^ and *P. lanceolata*. Thus, this reverse calculation can be used as a methodical revisal, even if not all parameters are known, as long as there are valid references. However, this example also shows the importance of considering every methodological parameter for comparison of different studies. Due to the different radius, the results of our two experiments and those of Huth et al. (2022)^29^ are not directly comparable, as both force and SF still differ about 10%, which is higher than the methodological uncertainty in our Experiment 1 (3%).

Another effect, which needs to be considered is the angle of the substrate to the centrifugal force vector. The assumption that the adhesion force equals the centrifugal force at the moment of detachment and can therefore be measured directly, is actually true only for an angle of 90°. As soon as particles are able to slide on an angular surface, a friction component occurs. A theoretical calculation shows that the combined effect of the force angle and the friction coefficient of the particle can reduce the force necessary for detachment to 10% of the actual adhesion force that would be measured at 90°^36^. The angle in our experiment was 51°, indicating that the true adhesion force and SF at 90° might be different from our results. However, this condition was the same for all pollen grains and has no effect on the relative differences between the examined samples. On the other hand, the friction coefficient of different pollen on glass has not been examined yet to the best of our knowledge and therefore can not be excluded to affect the results. Further research might be able to address this question or just exclude the friction component by using a rectangular set up.

### Influence of sample handling and counting

In addition to the centrifugation, the handling of samples can also affect the results, as shown by our long time experiment. While the overall methodical error of sample handling did not exceed 5%, “constant rotation” with 1000 rpm generated less detachment during the first day than in “no rotation” samples, at least after round 5. Thus, the general detachment of pollen grains by a low centrifugal force must be treated with caution, because as the more sample handling occurs in the course of experiment, the correlation between centrifugal force and pollen detachment becomes less pronounced. However﻿, a statistical comparison to our other data on “fresh” *A. vulgaris* pollen proved the effect of sample handling to be neglectable in our “increasing rotation” experiment. Still, further data on other species and with a larger sample size should be analyzed to validate this conclusion.

Another effect which could not be totally avoided is the deposition of dust particles on the glass fragments, which needs to be considered during image analysis. Reliable counting is probably the most challenging part in quantitative analysis of micro particles, thus many software tools have been developed to provide an automatic procedure, diminishing the effect of observer bias. The particle analysis tool of ImageJ used in this study proved to be a suitable choice for our samples containing several hundreds of grains at the beginning, despite an only moderate resolution of images. However, in detail, this method appeared to be very sensitive to some features. The two parameters used for counting, namely the projected particle area and circularity, were both in the range of 6–50 pixel. Thus, even a difference of a few pixels could exceed the set range, making the number of counted particles dependent on the orientation of non-spherical pollen grains, as well as image brightness and contrast between grains and background. While this methodical error was the same for each sample, it was still individual for each pollen grain, implying a huge number of grains for valid results. As a consequence, the range of size and circularity needed to be set abundantly, which on the other hand enhanced the risk of including dust particles into counting, as described above for our long time experiment. In general, the number of grains in our study was large enough to compensate these potential errors, but some adjustments could be necessary for further studies on samples with minor number of grains.

### Pollen analysis vs. powder analysis

As mentioned before, there are to the best of our knowledge only few studies, which used centrifugation for pollen adhesion analysis^29,34^, and they did not discuss the methodological aspects and how they might affect the results. In the field of powder technology however, centrifugation is a well-established method^37^, which provides a valid quantitative characterization of particle-surface-interaction for many powder materials and different surfaces. A direct methodical comparison of powder and pollen adhesion analysis makes only limited sense. However, there are some fundamental aspects to consider. For example, Ibrahim et al. (2004)^38^ stated that at least 30–40 particles per sample are necessary to statistically accurately perform adhesion analysis. Felicetti et al. (2008)^39^ stated that 2 min of centrifugation are sufficient, as a longer duration has no additional effect on the observed adhesion. You and Wan (2014)^40^ described a methodical error of 7% for their experiments due to slight variation in centrifugal radius and speed. As it can be seen from our results, our data fits very well into these criteria and, interestingly, despite the described differences, a range of adhesion force in our experiments is rather comparable to the literature data, as for example the analysis of starch and lactose particles^31^.

### Influence of individual contact conditions on the adhesion

Despite the majority of grains in each sample detached during the first rounds, there was always a small number of individual grains, which resisted even the highest centrifugal force. While studies on inanimate powders describe such a distribution as size dependent^31^, pollen of one sample are rather similar in size, proving other factors, such as their surface properties to be essential for their individual adhesion and SF. The work of adhesion can be described as the work necessary to separate a unit area of two surfaces from contact to infinity^41^, thus aside from friction, interlocking and fluid coating, the size of the actual contact area is critical for the strength of the required force. The actual contact area depends on the position and orientation of pollen grains on the substrate and contributes to the observed range of SFs. However, in a real situation, it is difficult to predict, whether the contact area varies enough to solely cause the observed variation in adhesion. Detailed measurements of the contact area of individual grains could help to answer this question.

Also, the distribution of surface roughness across the substrate can also affect the local conditions for adhesion of individual pollen grains. While the glass fragments that were used as substrates can be described as rather smooth in relation to the pollen grains, even fluctuations in a nanoscale range can possibly affect local adhesion^40^. Statistical analysis on the six “fresh” *A. vulgaris* samples showed strong significant differences between the samples. Thus, the composition of each sample, the amount of grains and their random orientation, which affects the actual contact area, indeed cause strong natural variation in the observed adhesion force distribution that can possibly obscure the effect of species-specific properties. As a consequence, statistical analysis with a low number of replications must be treated with caution, despite a huge amount of pollen grains in total.

### “Fresh” pollen adhesion differs between plant species

The differences among “fresh” pollen are not extraordinary strong in the range of low SF (round 1–3), indicating that the majority of grains shares rather similar adhesion conditions, despite belonging to different species. Instead in the range of high SF (round 4–10 and remaining), the two species, *H. radicata* and *P. sylvestris* show notably more attached grains that the others. For *H. radicata* this can probably be explained by the additional contribution of pollenkitt, also leading to the higher amount of remaining grains, compared to all others. The good adhesion of *P. sylvestris* instead was rather unexpected, as the grains are irregularly shaped with two large air-filled sacs and at the same time show less ornamentation than for example *A. vulgaris* grains, implying inferior conditions for attachment in general (Fig. 2). In addition, the pollenkitt is completely lacking in gymnosperms^15^. On the other hand, the surface of dry *P. sylvestris* grains is rough only at a nanoscale (Fig. 2), probably generating stronger adhesion on a glass surface compared to microscale ornamentation in other plant species. One study described pollen of pine species, namely *Pinus mugo* Turra to be covered with a thin layer of epicuticular waxes that makes at least 1.3% of the dry pollen weight^42^. Considering this amount of waxes, they may also affect mechanical surface-surface interaction on a nanoscale and probably contribute to the large number of grains with a high SF. Additionally, the large grain size, implying larger contact area, in combination with a low density may contribute to stronger adhesion^43^.

The three other species (*A. vulgaris*, *L. perenne* and *P. lanceolata*) show a distribution of “fresh” pollen that is rather similar to each other, with a slight tendency of lower adhesion in the stated series of species. The stronger adhesion of *A. vulgaris* compared to *L. perenne* possibly correlates with the smaller grains size of *A. vulgaris*, assuming a similar density. Considering the volume of dry grains estimated from Fig. 2, our weight measurements provided a density of 1.174 g/cm^3^ for *A. vulgaris* and while we did not measure the weight of *L. perenne* pollen, one previous study described a density of 1.140 g/cm^3^ for pollen of various corn species^44^.

In this context, the grain size of both species can be regarded as a critical factor for different adhesion. In addition, the slightly more pronounced ornamentation of *A. vulgaris* grains might also contribute to this result. In the same line, the lower adhesion of *P. lanceolata* can be explained, as grain size, shape and ornamentation are close to *L. perenne* (Fig. 2). The very low adhesion possibly also depends on the contact area due to deformation of dry pollen grains. SEM analysis on *P. lanceolata* of our own showed the strong effects of pollen-drying in this species (Fig. 2E, F). In addition, previous studies provide SEM images which show pollen of several *Plantago* species in different stages of crumpling^45^, indicating that the actual contact area might be very small in relation to the fresh pollen grain size.

The genus *Plantago* is a good example for ambophily, with many species representing different stages of adaptation to insect or wind pollination. Hesse (1979)^46^ examined pollen of three *Plantago* species, including a simple adhesion experiment through detaching grains from a glass slide by only gravity, gentle percussion or gentle blowing. The results described *P. lanceolata* as the most well adapted to anemophily among the three species. Interestingly, the pollen examined in that study showed rather well adhesion in the beginning, but rapidly lost their adhesion to the point, at which all grains were detached by gentle blowing. *P. lanceolata* pollen contain small amounts of pollenkitt, but it is inhomogeneous and clumpy and obviously does not provide strong adhesion^46^. Our results confirm well these data leading to the conclusion that the adaptation of *P. lanceolata* pollen to distribution by wind is at least as well as that of *A. vulgaris* and *L. perenne*, despite its ambophilous character.

### Decrease and increase of “aged” pollen adhesion

The results on “aged” pollen in contrast to “fresh” ones showed time-dependent changes for all examined species. Pollen of *H. radicata* and *P. sylvestris* followed a similar trend to lower adhesion, despite their completely different surface structures. In *H. radicata* this phenomenon was already described^29^, but adhesion of “aged” pollen in this species was the highest in comparison to all other species studied. Interestingly the second most adhesive species in “fresh” condition, *P. slyvestris*, showed strong decrease in adhesion with the aging time, becoming the least adhesive species in “aged” condition. The main contribution may probably be changes in the amount of epicuticular waxes. Thus, one can assume that pollen without waxes (washed in organic solvents) might lead to different results. In general, aging seems to have a similar effect on *P. sylvestris* pollen like on *H. radicata*, but it is much more pronounced in *P. sylvestris*.

In contrast, “aged” pollen of the other three species showed even stronger adhesion than “fresh” ones, at least to some extent. These observed changes were not similar for all regarded ranges of SF, as described in the results section. For *L. perenne* and *P. lanceolata*, the percentage of grains with moderate SFs decreased for the benefit of both, weak and strong adhesive grains. Whether this was a consequence of opposite effects of aging or just due to natural variation can not be confirmed at the moment. In general, an increase of adhesion was rather unexpected, but maybe water loss, leading to a more viscous pollenkitt, is responsible for stronger adhesion due to the contribution of viscous forces^29^. However, in species without a continuous layer of pollenkitt coating, the reasons for the adhesion increase might be different.

Like in *P. lanceolata*, also pollen grains of *A. vulgaris* contain small, inhomogeneous amounts of pollenkitt in exine cavities^46^. Previous studies described for most grasses a complete reabsorption of coating materials and therefore a clean surface^47^. Still their exine is a complex three-dimensional structure with micropores^48^, which probably contain some residues of coat. Changes in chemistry due to aging might enhance the stickiness of these residues and cause them to reappear on the surface, resulting in the slightly enhanced adhesion of “aged” grains. Nevertheless, further examinations with AFM are necessary to understand this tendency.

### Pollen aging “in contact” are cemented to the surface by the Pollenkitt

For pollen of *H. radicata* that aged “in contact” with the glass substrate, a strongly enhanced adhesion was previously described^29^. A comparison to our results showed that this increase was indeed far stronger than for all examined anemophilous species, clearly demonstrating the cementing effect of abundant pollenkitt. Interestingly, a similar, though less pronounced effect can be seen for *A. vulgaris*, which even surpasses *P. sylvestris* as the second most adhesive species. This effect might also be caused by the residues of pollenkitt. Stickiness of *A. vulgaris* pollen is almost always low, but can be notably higher in some specimens, as strong differences in pollenkitt production and distribution appear between individuals^46^. As mentioned before, the sample size of plant individuals in our study was rather low. Therefore, an effect of individual effects on our results can not be excluded, making further analysis of individual variability necessary, especially in the case of *A. vulgaris*. On the other hand, *P. sylvestris* showed a surprisingly strong difference to both, “fresh” and “aged” pollen, which might be caused by the presence of abundant wax layer. Interestingly, this change mainly occurred in the range of moderate SFs, as both, detachment in the first round and also number of remaining grains were similar to “fresh” ones, indicating that neither the weakest nor the strongest grains in contact were affected by aging, referring to their SFs. Especially grains with a high SF might represent some special contact conditions and therefore be affected in a different way than the others. A more detailed future analysis would be necessary to answer this question.

For *L. perenne*, there was only a slight tendency to more grains with a high SF, which can possibly be explained by the presence of residues of pollenkitt. In contrast, grains of *P. lanceolata* “in contact” showed a similar distribution as “fresh” ones and even less adhesion than “aged” pollen. While this result still must be properly validated, it indicates that additional processes might compensate the positive effect of aging on stickiness. As mentioned before, dry pollen of *P. lanceolata* tend to crumple. This might probably lead to a general reduction of contact area for all grains “in contact”, while “aged” grains still have the opportunity to land in a “lucky” position, regardless of their shape. In this case, the composition of the sample as a whole might cause the observed tendencies.

### Detailed analysis on *A. vulgaris*

The detailed examination of *A. vulgaris* grains helped us to further improve our centrifugation method of testing. Weight measurements of a high amount of particles, in combination with visual observation and counting, is an established method to determine the mass of micrometer sized particles and delivered valid results in previous studies for inanimate powders^30^ as well as for pollen grains^29^. The mass of 4.976 ng and 4.919 ng for “fresh” and “aged” *A. vulgaris* pollen, respectively, fits well to previous results on other species, which described mass values ranging from 3.43 ng (*Salix caprea* Linné) to 21.95 ng (*P. sylvestris*), with respect to different grain volumes^27,49,50^.

The neglectable difference between our both weight series indicates that the grains keep a similar water content over at least seven days. In fact, plant pollen grains are generally described to be released in a dry state, loosing most water before anther opening. However, this also differs, as for example Poaceae and *Plantago* species have a higher water content of more than 30%^51^. Weight measurements on those species might therefore reveal a significant difference between “fresh” and “aged” grains that needs to be considered during force measurements. Furthermore, our range of calculated adhesion force from 3.560 nN to 360.076 nN also fits well to the previous results^23,29^, as well as to several studies on inanimate micro particles (see Table 1 in Chen et al., 2018^52^), validating our results despite the potential error sources previously discussed.

The measurements over time, considering “2 h”, “1 day” and “7 days” indicate a rather steady change of adhesion. For “aged” grains, maximal adhesion was observed after 1 day, which means that the stickiness of free pollen grains was most effective at this point and decreased again afterwards. Interestingly, the percentage of moderately and highly adhesive grains was even higher than for grains “in contact” at the same stage, indicating a better initial contact formation to the substrate compared to long contact. However, what caused this phenomenon remains unclear. Also, we only analyzed one single sample of “1 day” “aged” pollen grains, which might be not a sufficiently representative result. In contrast, the adhesion of grains “in contact” increased even further and was the highest after 7 days.

Assuming that like in *H. radicata* the residues of pollekitt are indeed responsible for the time-dependent increase, a comparison of both categories probably demonstrates the cementing effect^29^. In this case, the properties of aging pollenkitt would enhance the stickiness to the age of at least 1 day until viscosity becomes too high to form a new bonding to the surface, while an already formed bonding would be further reinforced. Also, the number of remaining grains “in contact” stayed rather low for the first “2 h” and increased afterwards. This might indicate, that the bonding process needs at least two hours to become effective. More comparative detailed time-dependent analysis on other species needs to be done in the future to test this hypothesis also with respect to the dehydration rate and viability of grains. For example, grass pollen grains are known to be viable only for a few hours^53^ and might show different behavior within this rather short time. However, at this point, our method is limited due to the fact that one measurement series already took about 2 h, making it impossible to test shorter times of the same sample simultaneously with only one centrifuge.

### Conclusions and outlook

The aim of this study was to establish the mass centrifugation setup for quantitative analysis of pollen grains and to provide comparison of pollen adhesion between plant species with different pollination syndrome. Our results show that this method can be used to distinguish the pollen adhesion distribution in different plant species, even if the total range of adhesion is rather similar. Furthermore, changes in adhesion over time due to pollen aging can be determined. The effect of high amounts of pollenkitt clearly separates *H. radicata* from all other species, underlining its entomophilous ecology. The pollen grain surface of *P. sylvestris* shows surprisingly remarkable changes between all three categories of grains studied, indicating that the layer of epicuticular waxes in and on the exine has a similar effect on adhesion as the pollenkitt in angiosperms. The SF-distribution among the other three species clearly fits to their anemophilous ecology. However, aging and especially aging “in contact” reveals some rudiments of their entomophilous ancestry, mainly the presence of residuals of the pollenkitt. Interestingly, this was most pronounced for *A. vulgaris* and least pronounced for *P. lanceolata*. Therefore, in a final ranking considering all 15 settings (Fig. 5), the two Asteraceae species show the strongest adhesion, representing a good example of the opposite effects of genetic background on one side and ecology on the other side. Furthermore, our study highlights the role of several factors, such as centrifugal radius and angle, pollen grain orientation, surface roughness, sample handling, imaging details and counting approach, which are crucial for proper use of the centrifugation method of pollen adhesion characterization.

As we only compared the adhesion properties of pollen grains on glass, our study does not provide new results on pollen-plant-interaction so far. Natural surfaces are highly variable and even surfaces made of the same material but especially the body surface of pollinators can remarkably differ in their roughness and surface chemistry, making a comparable analysis of different species in a natural setup difficult if not impossible. In addition, varying surrounding conditions such as local temperature and humidity are further factors affecting pollen-plant-interaction in vivo. Thus, a standard method under standard conditions is necessary to provide comparable data about pollen grain adhesive properties that can be used as a basis for formulation of further hypotheses. Our centrifugation results provide information about the forces that are necessary to detach a single grain only due to its own mass and surface properties, enabling us to predict the detachment probability under certain natural conditions like wind gusts^27^ or vibration^35^. With this knowledge, we can argue, how the surface properties of the substrate affect the overall adhesion and how plants get evolved their stamen and stigma surfaces, to enhance or reduce the detachment probability of pollen grains. Furthermore, it is also generally important to consider the adhesion properties of pollen grains also on artificial surfaces in various other contexts, including allergenicity^54,55^, surface contamination^56^ and even forensics^57,58^, and artificial pollination aspects^59,60^.

Our research is an attempt to fill the gap of knowledge about the role of physical forces in the pollination process and provide perspectives of how to ensure successful plant pollination facing the challenges of climate change and loss of pollinators. In the future, a combination of the centrifugation method with other methods, such as AFM analysis and 3D modelling can help to precisely address the influence of species-specific properties (exine microstructure, grain deformation/crumpling, degree to pollenkitt coating) on the adhesion properties. Follow-up studies could also consider other substrates besides glass, as well as pollen grains with and without their coatings. Another goal should be to measure adhesion directly on stamen and stigma surfaces, to directly put the attachment behavior into the context of release and capture strategies and to investigate, how they are affected by changing environmental conditions.

## Electronic supplementary material

Below is the link to the electronic supplementary material.

Supplementary Material 1

Supplementary Material 2

## Data Availability

All data samples collected by our own are provided via the Supplementary Materials. Original data of *H. radicata* samples were taken from: Huth S. (2022). 2022-07 Data Quantifying the influence of pollen aging on the adhesive properties of *Hypochaeris radicata* pollen data sets. figshare https://doi.org/10.6084/m9.figshare.20238342.v1.
