# Supplementary material for: Time dependent attachment properties of pollen grains in anemophilous plants tested by the mass centrifugation method
Source: Sci Rep. 2025 Apr 29;15:15053. doi: 10.1038/s41598-025-99593-6 (PMC12041541; doi:10.1038/s41598-025-99593-6)
Supplement: Supplementary file 2 — Supplementary Material 2 [file 41598_2025_99593_MOESM2_ESM.docx]

**Time-dependent attachment properties of pollen grains in anemophilous plants tested by the mass centrifugation method**

**Supplemental materials**

**Martin Becker* and Stanislav Gorb**

Department of Functional Morphology and Biomechanics, Zoological Institute, Kiel University, Am Botanischen Garten 1–9, D-24118, Kiel, Germany

* corresponding author: mbecker@zoologie.uni-kiel.de

**Supplemental materials 2 – detailed evaluation of Table 1 and 2 and Figure 4 and 7**

**Copy of Table 1:** Summary of calculated safety factors from Experiment 1 and 2, referring to specific measurement rounds (see **Figure 4**).

| Comparison of all species - corresponding safety factors | | |
| --- | --- | --- |
|  | Experiment 1 | Experiment 2 |
| species | *A. vulgaris* | *P. lanceolata* |
|  | *L. perenne* | *H. radicata* |
|  | *P. sylvestris* |  |
|  | safety factors (SF 1) | safety factors (SF 2) |
| round 1 | < 73.779 | < 67.418 |
| round 2 | < 295.116 | < 269.673 |
| round 3 | < 664.010 | < 606.764 |
| round 4-10 | 664.010 – 7377.890 | 606.764 – 6741.826 |
| remaining | > 7377.890 | > 6741.826 |

**Copy of Table 2:** Summary of calculated maximal adhesion force for fresh and dry *A. vulgaris* pollen grains, referring to specific measurement rounds (see **Figure 7**).

| *Artemisia vulgaris* detailed analysis – maximal adhesion force | | |
| --- | --- | --- |
| categories: | fresh weight | aged weight |
|  | 4.976 ng | 4.919 ng |
|  | force [nN] | force [nN] |
| round 1 | < 3.600 | < 3.559 |
| round 2 | < 14.403 | < 14.238 |
| round 3 | < 32.407 | < 32.035 |
| round 4-10 | 57.612 - 360.076 | 56.952 - 355.951 |
| remaining | > 360.076 | > 355.951 |

**Detailed evaluation of Figure 4.**


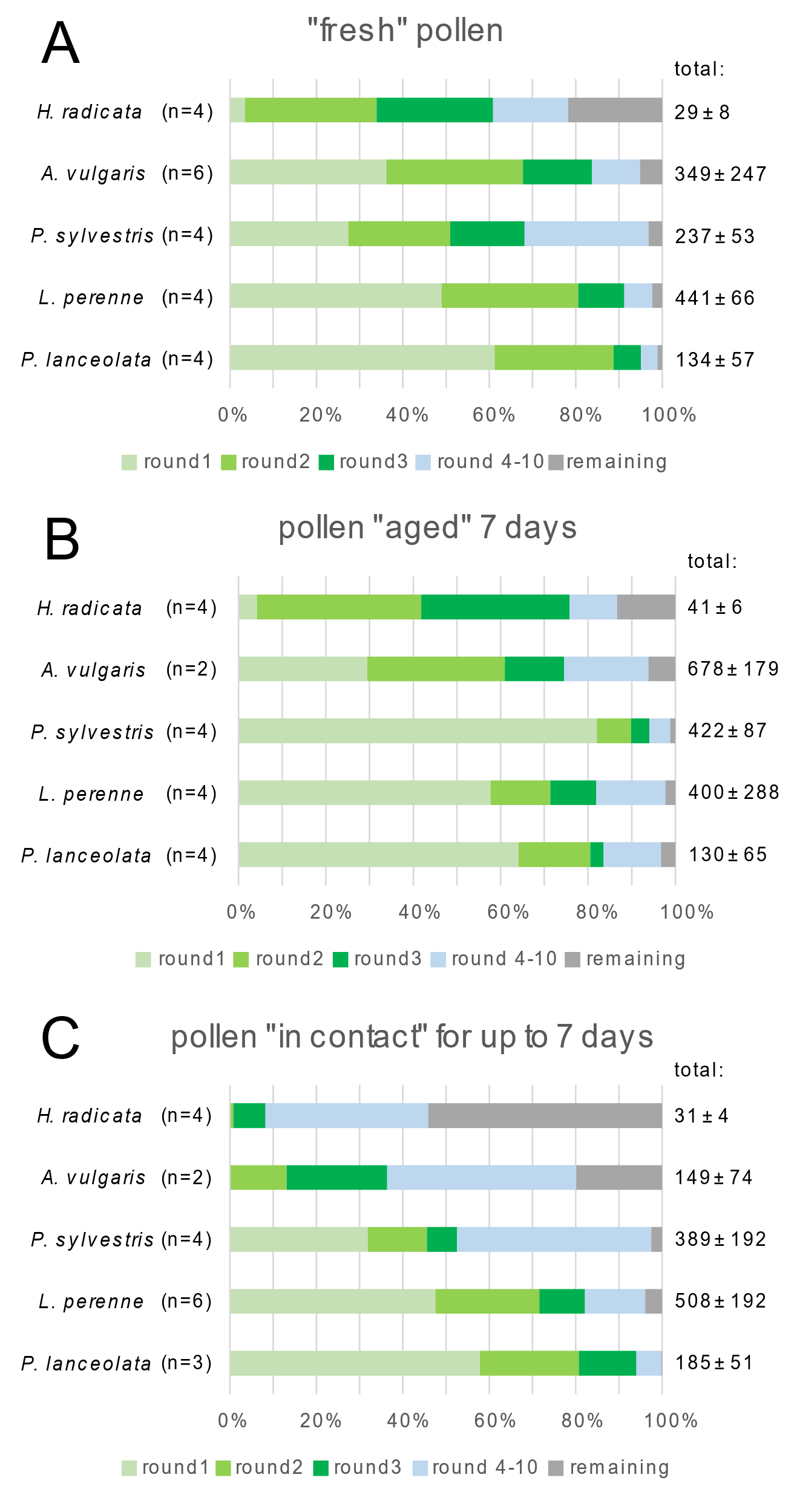


**Copy of Figure 4:** Summary of adhesion performance of pollen grains for all species and categories, showing the percentage of detached grains per round, which represent the distribution of measured adhesion safety factors (SF) in % for each category. **A:** “Fresh” pollen. **B:** “Aged” pollen. **C:** Pollen “in contact”. Corresponding safety factors are listed in **Table 1**. “Total” values represent the number of grains per category and species tested as mean and standard deviation, while “n” shows the number of replications.

**“Fresh” pollen**

Referring to fresh pollen of each species, the majority of pollen grains got detached within the first three rounds: 60 % of *H. radicata* pollen, nearly 70 % of *P. sylvestris*, 84 % of *A vulgaris* and more than 90 % of *L perenne* and *P. lanceolata*. However, there were strong differences between all species within the first rounds.

For *H. radicata*, only 3.5 % of pollen got detached in the round 1, while 30.4 % and 27 % got detached in rounds 2 and 3, respectively. For all other species, the strongest loss was already in the round 1, but the amount of grains varied strongly, beginning with 27.4 % for *P. sylvestris* and 36.2 % for *A. vulgaris*. For *L. perenne*, 49.0 % of pollen got detached in the round 1 and *P. lanceolata* showed the strongest loss, with 61.2 % in the first round. On the other hand, the detachment in the round 2 was rather similar for all species, with values ranging from 23.6 % (*P. sylvestris*) to 31.6 % (*L. perenne*). Also, for all species, the detachment in the round 3 and all following rounds was lower than in the round 2.

Another strong difference occurred in the number of grains that remained on the target area after the final round. *H. radicata* showed the highest amount of remaining grains with 21.7 %. This was followed with a huge gap by *A. vulgaris* with 5.1 % and *P. sylvestris* with 3.2 %. Finally, *L. perenne* and *P. lanceolata* had the lowest values of remaining grains with 2.3 % and 1.1 %, respectively.

As a consequence of these results, *H. radicata* among all species has the highest maximal safety factor (MSF) for fresh pollen, referring to the majority of grains, with a value of 223.6 and *P. lanceolata* has the lowest MSF of 67.4. The majority of grains of the three remaining species detached in the first round with a slightly higher MSF of 73.8, which corresponds to a G-force of 74. However, this result was most pronounced for *L. perenne* and fewest for *P. sylvestris*. Also in general, over the whole range of increasing SF, pollen of *H. radicata* showed the best adhesion, followed by *P. sylvestris* and *A. vulgaris*, while the SF distribution in *L. perenne* and *P. lanceolata* was obviously shifted to lower SFs. The overall adhesion ranking for all species and categories is summarized in **Figure 5**.

**“Aged” pollen**

For the category “aged”, pollen of the two species *H. radicata* and *P. sylvestris* tended to lower adhesion and increasing detachment during the first three rounds in comparison to “fresh” pollen. In detail, 75.8 % of *H. radicata* pollen and 94.0 % of *P. sylvestris* pollen detached during the first three rounds. On the other hand, the percentage of detached grains per round 1-3 slightly decreased for *A. vulgaris* to 74.5 %, for *L. perenne* to 82 % and for *P. lanceolata* to 83.6 %.

Detachment in the round 1 also showed some differences. Especially, *P. sylvestris* showed a remarkable loss of 82 %, which was about three times higher than for “fresh” pollen and also the highest loss among all species in this category. The other species showed less intense differences. *H. radicata* again had the lowest loss with only 4.2 % in contrast to 37.6 % in the round 2 and 34 % in the round 3. *A. vulgaris* showed a slight decrease to 29.5 % in the round 1, while *L. perenne* and *P. lanceolata* slightly increased to 57.7 % and 64.1 % respectively. As a consequence, the values in the round 2 changed as well, with a decrease for *P. sylvestris, L. perenne* and *P. lanceolata* and a slight increase for *A. vulgaris* and *H. radicata*. For *A. vulgaris* also the highest loss in one round shifted from the round 1 to the round 2 where 31.4 % loss was observed. Again, for all species, detachment in the round 3 and all following rounds was lower than in the round 2.

The number of remaining grains slightly increased for *P. lanceolata* (3.3 %) and *A. vulgaris* (6.2 %) and remained the same for *L. perenne* (2.3 %). For *P. sylvestris*, the number of remaining grains slightly decreased, resulting in the lowest value of 1.2 %, and it remarkably decreased for *H. radicata*, which still had the highest amount of remaining grains with 13.3 %.

Referring to the distribution across the safety factors, it can be concluded that the three species *P. lanceolata, A. vulgaris* and *L. perenne* slightly shifted their SFs to higher values and stronger adhesion, while *H radicata* slightly and *P. sylvestris* dramatically shifted them to lower adhesion. However, the absolute values of MSF for the majority of grains remained the same as for “fresh” pollen, except for *A. vulgaris*, as for this species the majority of “aged” grains detached with a higher MSF of 295.1 in the round 2.

**Pollen “in contact”**

The category “in contact” showed the strongest difference overall, compared to both “fresh” and “aged” pollen and this was more or less pronounced for each species. For *P. lanceolata*, the distribution of grains “in contact” was more similar to “fresh” pollen than to “aged” one, as 94.0 % of them detached during the first three rounds. In *P. sylvestris* and *L. perenne* all three categories were equally different from each other, while this difference was much stronger in *P. sylvestris*. In detail, only 52.5 % of *P. sylvestris* grains detached during the first three rounds, which was less than for “fresh” pollen and much less than for “aged” one. For *L. perenne*, more than 80 % of grains detached in rounds 1-3, but with some differences in each round. *H. radicata* showed the strongest difference with only 8.2 % loss in the first three rounds, directly followed by *A. vulgaris* with 37.2 %. For both these species, this was much less detachment than for “fresh” and “aged” pollen. Also, for both these species, there was no detachment at all in the round 1 and the strongest detachment shifted to the round 3 with 7.4 % in *H. radicata* and 23.8 % in *A. vulgaris*.

For the other three species, the strongest detachment was still in the round 1. Compared to “fresh” pollen, it was slightly less for *P. lanceolata* (57.8 %) and *L. perenne* (47.5 %) and slightly more for *P. sylvestris* (31.9 %). As a consequence, detachment in rounds 2 and 3 was also less for *P. sylvestris* and *L. perenne*, while that in *P. lanceolata* was slightly shifted to more detachment in the round 3. However, for all three species, detachment was still stronger in the round 2 than in 3.

The percentage of pollen detached at higher forces during rounds 4-10 also remarkably increased for *P. sylvestris* (45 %), *A. vulgaris* (44.8 %) and *H. radicata* (37.7 %). For *L. perenne* (13.9 %) and *P. lanceolata* (5.8 %), this percentage was far less pronounced. Also, it was higher than that for “fresh” pollen, but lower than that for “aged” ones. Referring to the remaining grains, *H. radicata* had by far the highest amount of all with 54.1 %, followed with a large gap by *A. vulgaris* with 20.3 %. For the other three species, the number of remaining grains stayed lesser than 5 %, like it was already reported above for “fresh” and “aged” pollen.

For the safety factors, one can conclude that for *L. perenne* and *P. lanceolata* there was only a slight difference in comparison to the other categories. For *P. lanceolata*, pollen “in contact” showed even lesser attachment than “aged” ones. However, for the other three species, adhesion strongly increased. Almost 50 % of *P. sylvestris* and more than 60 % of *A. vulgaris* pollen had safety factors of more than 664.0, which corresponds to a G-force of 664. For *H. radicata*, over 90 % of pollen had SFs of more than 606.8 and more than 50 % had SFs beyond the maximal centrifugal acceleration (> 6741.8). Also 20 % of *A vulgaris* pollen had a safety factor of more than 7377.9, marking grains of these two species as the most adhesive ones overall, followed by *P. sylvestris, L. perenne* and finally *P. lanceolata* as the least adhesive one (see **Figure 4** and **5**)

**Detailed Evaluation of Figure 7**


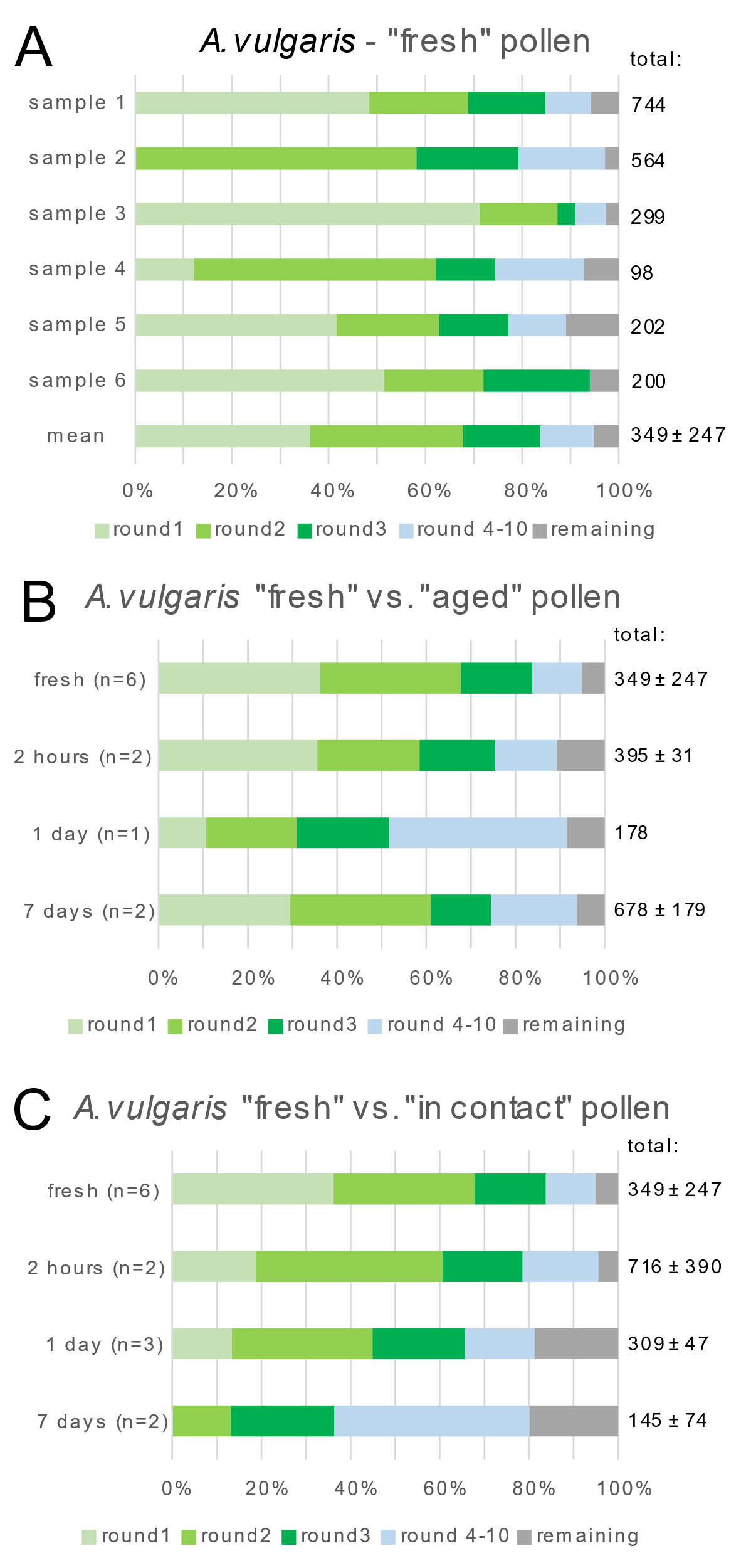


**Copy of Figure 7:** Summary of adhesion performance of pollen grains from detailed experimental analysis on *A. vulgaris*, showing the percentage of detached grains per round, which represent the distribution of measured adhesion forces and safety in % for every setting. **A:** Summary for “fresh” pollen. **B:** Summary for “aged” pollen. **C:** Summary for pollen “in contact”. Corresponding adhesion forces are listed in **Table 2**. “Total” values represent whole number of grains per setting as mean and standard deviation, while “n” shows the number of replications.

**Statistical analysis of “fresh” *A. vulgaris* pollen**

In addition to considering the mean value for comparison of species, the six samples containing “fresh” *A. vulgaris* pollen were compared statistically by counting the measured maximal adhesion force and the maximal adhesion SFs respectively of every single pollen grain as single values and therefore considering each sample separately. Following this approach, the sample 1 had the highest total sample size of 744 pollen grains (n=744). Furthermore, we got the sample 2 with n=564, the sample 3 with n=299, the sample 4 with the lowest sample size of n=98, the sample 5 with n=202 and the sample 6 with n=200.

In detail, the distribution of detachment over 10 rounds, as described above for all species, showed strong variation among the single *A. vulgaris* samples, ranging from 0 % to 70 % in the round 1, from 16 % to 60 % in the round 2 and 4 % to 22 % in the round 3. Nevertheless, the great majority of grains in all samples detached during the first three rounds, as only 0 % to 18 % detached during rounds 4-10 and 3 % to 7 % in total remained after the round 10. The results are summarized in **Figure 7 A**.

**Measurements over time in *A. vulgaris***

The detailed measurements of *A. vulgaris* over time, summarized in **Figure 7 B** and **C**, revealed some further differences due to pollen age. Referring to “aged” pollen, all three sub-categories “2 h”, “1 day” and “7 days” showed less detachment in the first three rounds than in “fresh” pollen (83.7 %). However, there was no clear tendency over time, as detachment was lowest after “1 day”, but increased again after “7 days”. In detail, 75.3 % of “2 h” old pollen got detached during round 1-3, compared to only 51.7% after “1 day”, but again 74.5 % after “7 days”. As a consequence, the number of detached grains during rounds 4-10 also changed and was highest for “1 day” with 39.9% followed by “7 days” with 19.2 % and 13.9 % for “2 h”, compared to only 11.1 % for “fresh” pollen. The amount of remaining grains slightly increased in the first place from 5.2 % (“fresh”) to 10.8 % (“2 h”), but decreased again after “1 day” (8.4 %) to finally 6.2 % after “7 days”.

Therefore, it can be said that the general adhesion force of “aged” *A vulgaris* pollen increased during the first 24 h, as almost 50 % of “1 day” old grains got detached by a force of more than 57.0 nN, compared to only 16 % of “fresh” pollen. Already after “2 h”, this percentage increased to nearly 25 %, but after “7 days” it decreased again to 26%. The highest adhesion showed almost 11 % of “2 h” old remaining grains that even resisted the maximal centrifugal force of 356.0 nN, which corresponds to a G-force of 7378.

The pollen “in contact” have rather remarkable differences to “aged” ones. There was a clear tendency of increasing adhesion over time, as 78.5 % of “2 h” old grains detached during the first three rounds (compared to 83.7 % of “fresh” pollen), followed by 65.7 % after “1 day” and only 37.2 % after “7 days”. Also, the amount of grains that detached in the round 1 strongly decreased from 36.2 % (“fresh”) over 18.8 % (“2 h”) and 13.4 % (“1 day”) to finally 0 % after “7 days”. Referring to rounds 4-10, the amount of detached grains only slightly varied during the first 24 h, but strongly increased after “7 days” to 44.8 %. In addition, the number of remaining grains already increased after “1 day” to 18.8 %, but stayed the same even after “7 days” (20.3 %).

As a consequence, the general adhesion force for pollen “in contact” constantly increased over time and was strongest for “7 days” old pollen, as more than 60 % had an adhesion force stronger than 57.0 nN, compared to 34 % of “1 day” old pollen, 21 % of “2 h” old pollen and 16 % of “fresh” pollen. In addition, about 20 % of “1 day” old grains and “7 days” old grains, respectively, resisted the maximal centrifugal force of 356.0 nN. Therefore, we can conclude that pollen of *A. vulgaris*, which aged in contact for more than 1 day, show the strongest increase of adhesion.
